# FOXO3A Expression in Upper Tract Urothelial Carcinoma

**DOI:** 10.3389/fonc.2021.603681

**Published:** 2021-04-20

**Authors:** Guoyao Zhang, Wanping Shi, Enzhao Jia, Lei Zhang, Yongsheng Han, Ronald Rodriguez, Tianjiang Ma

**Affiliations:** ^1^ Department of Oncology, Luohe Central Hospital, The First Affiliated Hospital of Luohe Medical College, Luohe, China; ^2^ Department of Oncology, The Second Affiliated Hospital, Chongqing Medical University, Chongqing, China; ^3^ Department of Pathology, Luohe Central Hospital, The First Affiliated Hospital of Luohe Medical College, Luohe, China; ^4^ Department of General Surgery, School of Medicine, Qinghai University, Xining, China; ^5^ Department of Urology, University of Texas Health Science Center San Antonio, San Antonio, TX, United States

**Keywords:** FOXO3A, prognosis, overall survival, recurrence-free survival, upper tract urothelial carcinoma

## Abstract

**Background:**

Epidemiological studies have reported various results regarding whether FOXO3A is related to various carcinomas. However, the prognostic significance of FOXO3A in upper tract urothelial carcinoma (UTUC) remains unclear. The purpose of this study was to validate the correlation between FOXO3A expression and oncological outcomes in UTUC.

**Methods:**

The expression levels of FOXO3A in 107 UTUC patients were examined by immunohistochemistry (IHC). We examined the prognostic role of FOXO3A by using the Cox proportional hazard model.

**Results:**

The results indicated that FOXO3A expression was notably decreased in UTUC tissue compared with control tissue. Decreased expression of FOXO3A was also related to advanced pathologic stage (*P* = 0.026), lymph node metastasis (*P* = 0.040), lymphovascular invasion (*P* < 0.001), and adjuvant therapy (*P* = 0.048). In addition, UTUC patients with low FOXO3A expression had a significantly shorter survival time, including both overall survival (OS) [hazard ratio (HR) 2.382, *P* = 0.004] and recurrence-free survival (RFS) (HR 2.385, *P* = 0.004), than those with high expression. Multivariate analyses showed that FOXO3A was a significant predictor for OS (HR 2.145, *P* = 0.014) and RFS (HR 2.227, *P* = 0.010) in UTUC patients.

**Conclusion:**

Our results indicate that FOXO3A may be involved in the recurrence of UTUC and that it has certain clinical value in the therapeutic targeting and prognostic evaluation of UTUC.

## Introduction

Urothelial carcinoma (UC) is the most common malignancy of the urinary tract. However, upper tract (renal pelvis and ureter) tumors account for only 5% to 10% of all UCs, and almost 60% of upper tract UCs (UTUCs) are invasive at diagnosis (1, 2). Currently available prognostic tools that utilize clinical and pathological parameters are limited for UTUC. Through the analysis of biomarkers in pathological specimens, we may strengthen the risk stratification and guide better prognostic evaluations for a more effective therapeutic strategy (3–5).

Forkhead box O 3a (FOXO3A) belongs to the FOXO protein family and is located on human chromosome 6q21 (6). FOXO3A activity and stability can be regulated by post−translational modifications, including phosphorylation, acetylation, ubiquitination and glycosylation, aside from its well-validated modifications in transcription (7). It has been highlighted as an important transcriptional regulator of crucial proteins participating in DNA damage repair (8), cell cycle regulation (9), apoptosis (10), angiogenesis (11), and cellular stress response (12). The function and detailed molecular mechanisms of FOXO3A in tumor progression remain elusive. FOXO3A is downregulated and functions as a tumor suppressor in several types of tumors, including urothelial carcinoma. Downregulation of FOXO3A expression promotes tumor occurrence, metastasis, and progression in breast cancer (13), gastric carcinoma (14), pancreatic ductal adenocarcinoma (15), cervical carcinoma (16), clear cell renal cell carcinoma (17), and urothelial carcinoma (18).

In contrast, it plays a more complex supportive role in various types of malignancies. For example, in glioblastoma multiforme, overexpression of FOXO3A is positively correlated with tumor progression and predicts a poor survival outcome (19). Some studies have reported that activation of FOXO3A can lead to the elimination of cancer stem cells (20–22). FOXO3A also enhances the invasive ability of tumor cells by regulating matrix metalloproteinases in a number of tumor cell types (23, 24). However, whether FOXO3A serves as a useful biomarker in UTUC has not been reported.

Herein, we first detected FOXO3A by immunohistochemical analysis of UTUC patients and then investigated any potential association between FOXO3A and clinicopathologic parameters in patients with UTUC. Moreover, we examined the prognostic role of FOXO3A and aimed to build a predictive model for UTUC.

## Methods

### Patients

The present study was carried out at Luohe Central Hospital, Luohe, China. Ethical approval for this study was obtained from the Institutional Ethics Committee of the Department of Pathology, Luohe Central Hospital, Luohe, China.

We collected UTUC samples between November 2004 and December 2015 from the archives for immunohistochemical and survival analysis. Finally, 107 UTUC patients who underwent surgery in the pathology department of Luohe Central Hospital were selected. The inclusion criteria were as follows: 1) patients with a diagnosis of UTUC who underwent radical nephroureterectomy and had clinicopathological data, 2) patients for whom concomitant bladder cancer was excluded on cystoscopy, and 3) patients without any other malignancies or severe chronic disease.

Regular follow-up had no standard protocol due to the retrospective nature of the study. Patients received clinical and radiological follow-up in accordance with final pathology, guidelines at that time, and physician judgement. Clinical data were extracted from medical records, including tumor number, pathological stage, histology grade, depth of invasion, lymph node metastasis (LNM) status, lymphovascular invasion (LVI), histological differentiation, adjuvant therapy and neoadjuvant chemotherapy. The clinical features of the UTUC patients are listed in **Table 1**.

**Table 1:** Association between FOXO3A expression and UTUC clinicopathological parameters.

Parameter	Overall	FOXO3A (Low expression)	FOXO3A (High expression)	*P*-value
Patients (n)	107	67	40	
**Age, years**				
< 65	49 (45.8)	30 (44.8)	19 (47.5)	0.842
≥ 65	58 (54.2)	37 (55.2)	21 (52.5)	
**Gender**				
Male	71 (66.4)	42 (62.7)	29 (72.5)	0.398
Female	36 (33.6)	25 (37.3)	11 (27.5)	
**Multifocality**				
Multifocal	22 (20.6)	12 (17.9)	10 (25.0)	0.460
Single	85 (79.4)	55 (82.1)	30 (75.0)	
**Histologic grade**				
Low	37 (34.6)	27 (40.3)	10 (25.0)	0.142
High	70 (65.4)	40 (59.7)	30 (75.0)	
**pT stage**				
Ta/T1	46 (43.0)	23 (34.3)	23 (57.5)	0.026
T2/T3/T4	61 (57.0)	44 (65.7)	17 (42.5)	
**LNM**				
No	66 (61.7)	36 (53.7)	30 (75.0)	0.040
Yes	41 (38.3)	31 (46.3)	10 (25.0)	
**LVI**				
Absent	53 (49.5)	23 (34.3)	30 (75.0)	<0.001
Present	54 (50.5)	44 (65.7)	10 (25.0)	
**Histological differentiation**				
Pure urothelial	89 (83.2)	57 (85.1)	32 (80.0)	0.595
Variant histology^a^	18 (16.8)	10 (14.9)	8 (20.0)	
**Adjuvant therapy**				
Yes	32 (29.9)	25 (37.3)	7 (17.5)	0.048
No	75 (70.1)	42 (62.7)	33 (82.5)	
**Neoadjuvant chemotherapy**				
Yes	15 (14.0)	9 (13.4)	6 (15.0)	1.000
No	92 (86.0)	58 (86.6)	34 (85.0)	

LNM, lymph node metastasis; LVI, lymphovascular invasion; pT, pathological tumor stage.

a Variant histology included micropapillary, plasmocytoid, sarcomatoid, and neuroendocrine types.

### Immunohistochemistry

IHC staining for FOXO3a was performed following the manufacturer’s recommendations. Tissue section blocks were cut into 4-μm-thick slides, dewaxed and rehydrated. The antigen was recovered, and endogenous peroxidase activity was blocked. The slides were then incubated with anti-FOXO3a antibody (1:100 dilution; Abcam, Cambridge, UK) for 1 hour. The presence of brown chromophores in the nucleus and cytoplasm of target cells indicated positive immunoreactivity. Finally, the slide was examined by optical microscopy at 400× magnification. Negative and positive controls were scored to optimize staining.

### Evaluation of the IHC Results

FOXO3A-positive cells were assessed irrespective of the intensity of staining, and intracytoplasmic staining of FOXO3A was subsequently evaluated. The methodology applied by Tian et al. (16) was used to evaluate the FOXO3A score.

The percentage of cells positive for FOXO3A in the tumor stroma was recorded by two observers (TJM and LZ) as 0 = no positive cells, 1 = 1–10% positive cells, 2 = 10–50% positive cells, 3 = 50–80% positive cells, and 4 = >80% positive cells. The same score obtained by more than two observers was counted as the final score. Each specimen received a score of 0, 1, 2, 3 or 4 according to the intensity of FOXO3A staining. The product of the intensity score and stained area percentage was added and used as the total score. The final scores ranged from 0 to 8 and were designated low (0–4) or high (5–8).

### Statistical Analysis

The analysis of FOXO3A IHC staining between tumor tissue and adjacent normal tissues was assessed by the t-test or chi-square using GraphPad Prism 8 software. When FOXO3a IHC was deep sectioned, the tumor component of three of the 43 tumor tissues was lost, so the FOXO3a expression of these tumors could not be assessed; ultimately, these three tumor tissues were excluded from the analyses involving the tumor component expression. All 40 pairs of UTUC and adjacent normal tissues were considered in the remaining analyses.

The SPSS 26.0 software suite (SPSS Inc., Chicago, IL, USA) was utilized for all statistical analyses, and statistical significance was considered at *P* < 0.05. Associations between FOXO3A staining expression and clinicopathologic variables were estimated with Fisher’s exact test. Overall survival (OS) was defined as tumor-related death, and recurrence-free survival (RFS) was defined as any local recurrence or distant recurrence, whichever occurred first. The difference in OS and RFS between the FOXO3A-high and FOXO3A-low groups was assessed in univariable and multivariable settings. Variables such as FOXO3A expression, pathological tumor stage, and histological differentiation were grouped into two groups: high expression vs low expression of FOXO3A, early stage (Ta-T1) vs late stage (T2-4), and pure urothelial vs variant histology.

## Results

### Expression of FOXO3A Protein Is Decreased in UTUC Patients

**Figure 1:**
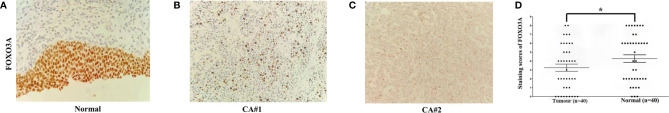
FOXO3a expression in UTUC tissues determined by immunohistochemical staining (original magnification, 200×). **(A–C)** Representative FOXO3a expression in tumor and normal tissues, with positive expression located in the nucleus. **(D)** Scatter dot plot showing the staining score (mean ± SEM) of FOXO3a in tumor and normal tissues using the paired t-test. *P<0.001.

Using the IHC staining method, the relative level of FOXO3A protein expression was assessed in 40 pairs of UTUC and adjacent normal tissues. Representative photographs of FOXO3A IHC are shown in **Figures 1A–C**. The scatter dot plot illustrated that the average immunoreactivity score of FOXO3A protein in 40 UTUC tissues was significantly downregulated compared with that of the 40 normal tissues (3.08 ± 0.43 VS 4.30 ± 0.44) (**Figure 1D**, *P <* 0.001). The expression of FOXO3A was lower in UTUC patients than in normal tissues (UTUC vs normal tissues: 13/40 vs. 22/40, *P <* 0.001).

### FOXO3A Immunoexpression Is Associated With the Clinical Parameters of UTUC Patients

The correlations of the relative FOXO3A expression level with clinical criteria are shown in **Table 1**. The results showed that low expression of FOXO3A was significantly associated with aggressive pathological stage (*P* = 0.026), lymphovascular invasion (*P* < 0.001), lymph node metastasis (*P* = 0.040), and adjuvant therapy (*P* = 0.048).

### Survival Analysis

**Figure 2:**
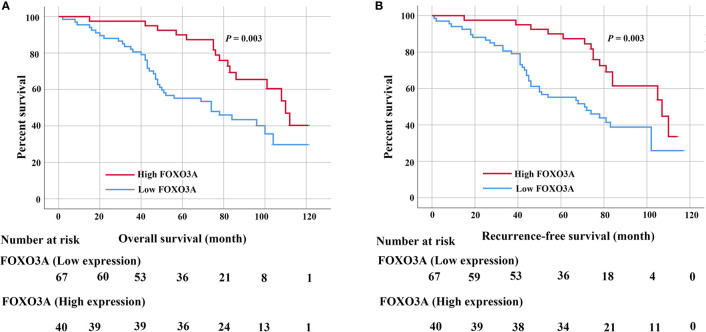
**(A)** Kaplan–Meier curves for the 5-year OS rate of patients with UTUC and OS based on FOXO3a in UTUC patients. **(B)** Kaplan–Meier curves for the 5-year RFS rate of patients with UTUC and RFS based on FOXO3a in UTUC patients. Error bar = SEM.

In the Kaplan-Meier analyses, patients with low FOXO3A expression had markedly worse OS (*P* = 0.003; **Figure 2A**) and RFS (*P* = 0.003; **Figure 2B**) than those with high FOXO3A expression. Furthermore, log rank tests demonstrated a significant difference between the 5-year OS (83.6% in the high expression group vs. 60.7% in the low expression group) and RFS (83.5% in the high expression group vs. 61.4% in the low expression group) rates in these two groups.

Univariate analyses determined that pathological stage (both *P* = 0.008), lymph node metastasis (both *P* < 0.001), lymphovascular invasion (both *P* = 0.028), adjuvant therapy (*P* = 0.025 and *P* = 0.015) and FOXO3a protein (both *P* = 0.004) were significantly correlated with both OS and RFS. Histologic grade (*P* = 0.049) was also associated with OS (**Tables 2**, **3**). However, only pathological stage, lymph node metastasis and FOXO3A protein expression were ultimately determined to be predictors of the OS (*P* = 0.031, *P* = 0.006, *P* = 0.014) and RFS (*P* = 0.022, *P* = 0.004, *P* = 0.010) of UTUC patients in the multivariate analyses (**Tables 2**, **3**).

**Table 2:** Univariate and multivariable analyses assessing the association between predictor variables and overall survival mortality among 107 patients for UTUC.

Characteristics	Univariate			Multivariate		
	Hazard Ratio	95%CI	*P-value*	Hazard Ratio	95%CI	*P-value*
**Age, years**						
≥ 65 *vs* < 65	1.110	0.649-1.894	0.704	0.875	0.482-1.588	0.660
**Gender**						
Male *vs* Female	0.699	0.404-1.207	0.199	0.754	0.428-1.328	0.329
**Multifocal upper tract disease**						
Multifocal *vs* Single	1.136	0.571-2.260	0.716	0.963	0.467-1.985	0.919
**Histologic grade**						
High *vs* Low	1.758	1.002-3.084	0.049	1.439	0.804-2.575	0.220
**pT stage**						
T2/T3/T4 *vs* Ta/T1	2.185	1.232-3.875	0.008	1.893	1.059-3.385	0.031
**LNM**						
Yes *vs* No	2.631	1.536-4.506	<0.001	2.152	1.245-3.721	0.006
**LVI**						
Present *vs* Absent	1.824	1.066-3.121	0.028	0.844	0.427-1.668	0.625
**Histological differentiation**						
Pure urothelial *vs* Variant histology^a^	1.017	0.497-2.081	0.963	0.870	0.406-1.867	0.721
**Adjuvant therapy**						
Yes *vs* No	1.887	1.084-3.282	0.025	1.203	0.662-2.187	0.543
**Neoadjuvant chemotherapy**						
Yes *vs* No	1.000	0.472-2.119	1.000	1.187	0.131-10.789	0.879
**FOXO3A expression**						
Low *vs* High	2.382	1.312-4.327	0.004	2.145	1.167-3.941	0.014

LNM, lymph node metastasis; LVI, lymphovascular invasion; pT, pathological tumor stage.

a Variant histology included micropapillary, plasmocytoid, sarcomatoid, and neuroendocrine types.

**Table 3:** Univariate and multivariable analyses assessing the association between predictor variables and recurrence among 107 patients for UTUC.

Characteristics	Univariate			Multivariate		
	Hazard Ratio	95%CI	*P-value*	Hazard Ratio	95%CI	*P-value*
**Age, years**						
≥ 65 *vs* < 65	1.100	0.645-1.877	0.726	0.876	0.490-1.567	0.655
**Gender**						
Male *vs* Female	0.671	0.386-1.167	0.158	0.713	0.403-1.263	0.247
**Multifocal upper tract disease**						
Multifocal *vs* Single	1.189	0.598-2.367	0.621	0.971	0.470-2.005	0.913
**Histologic grade**						
High *vs* Low	1.708	0.975-2.993	0.062	1.298	0.715-2.356	0.391
**pT stage**						
T2/T3/T4 *vs* Ta/T1	2.181	1.229-3.871	0.008	1.971	1.102-3.527	0.022
**LNM**						
Yes *vs* No	2.708	1.580-4.643	<0.001	2.257	1.306-3.900	0.004
**LVI**						
Present vs Absent	1.834	1.069-3.144	0.028	0.844	0.426-1.670	0.626
**Histological differentiation**						
Pure urothelial *vs* Variant histology^a^	1.055	0.515-2.165	0.883	0.860	0.397-1.865	0.703
**Adjuvant therapy**						
Yes *vs* No	1.991	1.142-3.471	0.015	1.402	0.779-2.521	0.259
**Neoadjuvant chemotherapy**						
Yes *vs* No	1.039	0.488-2.209	0.922	1.144	0.125-10.491	0.906
**FOXO3A expression**						
Low *vs* High	2.385	1.314-4.329	0.004	2.227	1.209-4.101	0.010

LNM, lymph node metastasis; LVI, lymphovascular invasion; pT, pathological tumor stage. ^a^Variant histology included micropapillary, plasmocytoid, sarcomatoid, and neuroendocrine types.

## Discussion

Most recent studies have investigated whether FOXO3A plays a key role in UC. Shiota et al. (18) showed that FOXO3A inhibits UC invasiveness *via* Twist1, YB-1, and E-cadherin regulation. Zhuo et al. (25) reported that upregulation of CSTP1 expression suppresses IL-6 expression by regulating the Akt/FoxO3a signaling pathway in UC. Zhu et al. (26) showed that ATG7 overexpression promoted autophagic removal of FOXO3A in bladder carcinoma cells. Nevertheless, little is known about the prognostic role and clinicopathologic implications of FOXO3A in UTUC.

The results of our study reveal the following: (1) low FOXO3A expression predicts unfavorable survival and recurrence rates in UTUC patients; (2) FOXO3A expression is negatively associated with pathological stage, lymph node metastasis status, lymphovascular invasion, and adjuvant therapy in UTUC patients; and (3) FOXO3A expression is lower in UTUC tissue than in normal tissue. These findings indicate that FOXO3A is a prognostic factor for UTUC and that adjuvant therapy may be helpful in the high-risk subgroup of UTUC patients.

The biological mechanism of FOXO3A also illustrates its key role in the pathogenesis of UTUC. FOXO3A is part of a subfamily of winged-helix transcription factors, and its activity can be regulated by PI3K/AKT signaling (6). FOXO3A has been identified as a tumor suppressor because of its ability to promote cell cycle arrest (27) and DNA damage repair (28) and to inhibit tumor cell properties and tumorigenesis (22). Interestingly, several potential substrates for correlations between FOXO3A and tumor metastases have been described in previous studies (15, 29). In addition, FOXO3A has been implicated in epithelial mesenchymal transition, an important process during metastasis, and downregulation of FOXO3A promotes tumor cell migration and invasion (30–32).

This study is the first to evaluate the associations between FOXO3A expression and clinicopathological features and prognostic factors in UTUC by IHC. FOXO3A can be considered an anti-oncogene, and overexpression or pharmacological activation of FOXO3A inhibits tumor progression and improves prognosis. A previous study confirmed that downregulation of FOXO3a expression is associated with poor prognosis in bladder carcinoma patients by RT-PCR. Based on these data, we suggest that FOXO3A expression in UTUC tends to indicate a good prognosis.

However, there are still limitations to the study. 1) UTUC is rare, and the sample size was relatively small. 2) Determination of FOXO3A status is limited to IHC detection of the protein without integrated methodology or a scoring system. 3) The detailed molecular mechanism of FOXO3A in UTUC remains unknown. 4) Because this is a retrospective single-center study, potential bias exists and cannot avoid confounding factors and the absence of a standard for follow-up assessment. Therefore, larger patient groups are needed to further investigate the role of FOXO3A in UTUC and help us to better understand the molecular events involved in the pathogenesis of UTUC.

There is a complex crosstalk between FOXO3a/AKT signaling pathway and PD-L1 involved in tumorigenesis (26, 33). PD-L1 is a critical regulator in UC development and the level of functional PD-L1 plays a vital role in the effective immunotherapeutic treatments for UC (34, 35). Therefore, further exploration of the relationship between FOXO3a and PD-1 may have important implications for UTUC immunotherapy. FOXO3a activity is directly regulated by some miRNAs in UC (26, 36). This suggests that finding or synthesizing new chemotherapeutic drugs targeting these miRNAs may also be a promising strategy for the treatment of UTUC.

In conclusion, our results showed that FOXO3A may be a potential biomarker for determining UTUC diagnosis and prognosis and may serve as a tumor-suppressing gene. Moreover, clarification of the underlying molecular mechanisms of FOXO3A in UTUC progression could aid in the development of targeted therapies for UTUC patients.

## Data Availability Statement

The original contributions presented in the study are included in the article/supplementary material. Further inquiries can be directed to the corresponding author.

## Ethics Statement

The studies involving human participants were reviewed and approved by The Institutional Ethics Committee of Luohe Central Hospital, Luohe, China. The patients/participants provided their written informed consent to participate in this study.

## Author Contributions

GZ, EJ, and LZ: writing and figures. GZ, WS, YH, RR, and TM: concept and proof reading. All authors contributed to the article and approved the submitted version.

## Conflict of Interest

The authors declare that the research was conducted in the absence of any commercial or financial relationships that could be construed as a potential conflict of interest.
